# Adherence to and enforcement of non-pharmaceutical interventions (NPIs) for COVID-19 prevention in Nigeria, Rwanda, and Zambia: A mixed-methods analysis

**DOI:** 10.1371/journal.pgph.0000389

**Published:** 2022-09-08

**Authors:** Hiwote Solomon, Donald M. Thea, Sandro Galea, Lora L. Sabin, Daniel R. Lucey, Davidson H. Hamer

**Affiliations:** 1 Doctor of Public Health Program, Boston University School of Public Health, Boston, Massachusetts, United States of America; 2 Department of Global Health, Boston University School of Public Health, Boston, Massachusetts, United States of America; 3 Department of Epidemiology, Boston University School of Public Health, Boston, Massachusetts, United States of America; 4 Department of Medicine, Georgetown University Medical Center, Washington, District of Columbia, United States of America; 5 Department of Medicine, Boston University School of Medicine, Boston, Massachusetts, United States of America; Management Sciences for Health, UGANDA

## Abstract

**Introduction:**

In the early parts of the COVID-19 pandemic, non-pharmaceutical interventions (NPIs) were implemented worldwide, including in sub-Saharan Africa, to prevent and control SARS-CoV-2 transmission. This mixed-methods study examines adherence to and enforcement of NPIs implemented to curb COVID-19 in Nigeria, Rwanda, and Zambia, leading up to the 10,000^th^ case of laboratory-confirmed COVID-19 in each country. Additionally, we aim to evaluate the relationship between levels and changes of NPIs over time and changes in COVID-19 cases and deaths.

**Methods:**

This mixed-methods analysis utilized semi-structured interviews and a quantitative dataset constructed using multiple open data sources, including the Oxford COVID-19 Government Response Tracker. To understand potential barriers and facilitators in implementing and enforcing NPIs qualitative data were collected from those involved in the COVID-19 response and analyzed using NVivo. Quantitative results were analyzed using descriptive statistics, plots, ANOVA, and post hoc Tukey.

**Results:**

Individual indicator scores varied with the COVID-19 response in all three countries. Nigeria had sustained levels of strict measures for containment and closure NPIs, while in Rwanda there was substantial variation in NPI score as it transitioned through the different case windows for the same measures. Zambia implemented moderate stringency throughout the pandemic using gathering restrictions and business/school closure measures but maintained low levels of strictness for other containment and closure measures. Rwanda had far more consistent and stringent measures compared to Nigeria and Zambia. Rwanda’s success in implementing COVID-related measures was partly due to strong enforcement and having a population that generally follow the recommendations of their government.

**Conclusion:**

Various forces either facilitated or hindered adherence and compliance to COVID-19 control measures. The lessons learned and recommendations gleaned through interviews with experts involved in the COVID-19 pandemic and quantitative analysis of NPI implementation can be applied to future outbreaks, epidemics, and pandemics. Recommendations include engaging communities, using a risk-based approach to implement containment and closure NPIs, and providing social and economic support to citizens during periods of lockdowns and other measures that interrupt the ability to make a living.

## Introduction

In a pandemic, non-pharmaceutical interventions (NPIs) are crucial in curbing disease spread, especially in the absence of vaccines and other pharmaceutical interventions [1]. It is widely accepted in public health that early interventions are an important step to halting the progression of a new communicable disease threat [2]. Prior to the COVID-19 pandemic, the impact of NPIs has been largely studied in controlling influenza outbreaks, including the 1918/1919 influenza pandemic [1, 3]. NPIs include actions that can be taken by individuals and the larger community. These include frequent hand washing, covering coughs and sneezes, isolating sick persons, contact tracing, quarantining exposed persons, and physical/social distancing measures for the general population [1, 4, 5]. Physical/social distancing measures include containment strategies such as the closing of schools and workplaces, restricting public gatherings, curfews, quarantine, and maximizing telework (when applicable) [5–8].

The success of NPIs is dependent on enforcement, political systems, and citizens’ willingness to comply with the measures. Until recently, the effectiveness of NPIs has not been tested systematically. Early measures of efficacy were explored mainly through the use of mathematical models, while few published studies have reviewed the historical evidence on NPI adoption during past pandemics [1, 3, 9]. A small number of studies have explored the impact of public health interventions and NPIs during the 1918–1919 influenza pandemic, in which NPIs played a critical role in delaying the temporal effects of the pandemic, in addition to reducing the overall and peak attack rate and the number of cumulative deaths [3, 9]. How successful NPIs were in limiting disease spread in Africa, especially in the first year of the COVID-19 pandemic has been underexplored in the literature. The degree of implementation and the impact of these NPIs during the COVID-19 pandemic has largely been studied in high-income countries, and there has been limited data in the literature focused on low- and middle-income countries, especially in Africa [10–15].

In the absence of treatment beyond supportive care and vaccination for the early parts of the COVID-19 pandemic, NPIs were implemented across the world to prevent and control the transmission and spread of SARS-CoV-2. By early March 2020, several countries in Africa (affected and unaffected by COVID-19) began mobilizing in response to the pandemic. This included prompt case identification, information campaigns to sensitize citizens, and building laboratory capacity [16, 17]. Some countries relied on innovative strategies such as using locally produced cloth masks, soaps, and hand sanitizers, developing inexpensive diagnostic tests, testing pooled COVID-19 samples, and using drones to transport test kits and samples to and from hard-to-reach areas [18–20]. By the end of March 2020, many African Union Member States had imposed travel bans on flights arriving from certain Asian and European countries [20]. In the following two months, almost two-thirds of African Union Member States had closed their borders to all international travelers, except for cargo, freight, and expatriation of foreign nationals [19, 20]. Fifteen countries, including Nigeria and Rwanda, implemented border closures before any COVID-19 cases were confirmed [21]. Other NPIs, such as restrictions of movement and public gathering, and closure of schools and workplaces were also implemented across the region.

Some countries replicated NPIs, such as travel restrictions and shelter-in-place orders, as implemented by Western countries in Europe and the United States. However, approaches that were effective in countries outside of Africa were not necessarily appropriate for the African region. Given the heterogeneity in populations, health systems, and governments in the region, blanket NPI measures such as restrictions on movement proved challenging to implement. Almost three-quarters (71%) of Africans work in the informal sector and thus encountered severe economic hardship with the enforcements of lockdowns and border closures [18, 22, 23]. With the implementation of certain measures, some countries had food shortages, social unrest, and economic instability [18, 19]. Economic instability was felt across the African continent; however, Zambia became the first African country to default on its Eurobond national debt during the pandemic [24]. The pandemic caused the Zambian economy to enter its deepest recession in history with the economy shrinking by 4.2% in 2020 [25–27]. An assessment of the Zambian economy a year into the pandemic claimed that the “recession goes beyond the containment measures (which were moderate) and reflects vulnerabilities to external shocks and unfavorable internal macroeconomic decisions, with potential long-term implications” [28]. Given the socioeconomic effects of implementing and enforcing NPIs, some countries were met with steep resistance from their citizens.

The present study focuses on three countries in Africa: Nigeria in West Africa, Rwanda in East Africa, and Zambia in southern Africa. These countries were selected to provide variety in geopolitical structures, population size, region, and World Bank income classification. This mixed-methods study aims to examine adherence and enforcement of NPIs implemented to curb COVID-19 in Nigeria, Rwanda, and Zambia, leading up to the 10,000^th^ recorded case of COVID-19 in each country. Additionally, we aim to understand facilitators and barriers related to compliance with NPIs in the three countries.

## Methods

### Study design

This mixed-methods study utilized a mix of semi-structured interviews and a quantitative dataset constructed using multiple open data sources.

### Qualitative method

To understand the potential barriers and facilitators in implementing and enforcing NPIs and how other epidemics within the countries may have affected compliance in NPIs, qualitative data were collected from decision-makers and experts involved in the COVID-19 response using key informant interviews (KIIs). KIIs were conducted with officials of Ministries of Health, Africa CDC Regional Collaborating Centers, and WHO African Regional Office (AFRO). Recruitment utilized purposive and convenience sampling, including a snowball sampling approach. All KIIs provided verbal consent before the start of the interview. Each KII took on average 30 minutes to complete using Zoom. The audio recordings were downloaded from Zoom and then immediately uploaded to a secure database and deleted from the computer. Transcription took place upon completion of the interviews. Data were collected and analyzed using a grounded theory approach [29]. Thematic analysis using inductive coding was used to systematically extract key themes in an iterative process as they emerged through the analysis process. An iterative process was used to develop a comprehensive codebook. During the initial coding phase, first-order codes were developed, while secondary coding allowed for the grouping of first-order codes into themes. Quotes attributed to specific themes were extracted. Both coding and analysis were conducted using NVivo Release 1.6 Mac Edition [30].

### Quantitative methods

A time-series dataset was constructed using multiple open data sources. Data sources are listed in Table 1. Observations for each variable were recorded daily beginning on January 1^st^, 2020, for each of the three countries (Nigeria, Rwanda, and Zambia) and ending on the date when each country surpassed its 10,000^th^ case. While most studies exploring the effect of NPIs have focused on 100 cases as the outbreak threshold, this study uses four case windows to gain a broader picture: no cases (W_0_), 1–100 cases (W_1_), >101–1,000 cases (W_2_), and >1,001–10,000+ cases (W_3_). New and cumulative cases during this period were obtained from the COVID-19 data repository by the Center for Systems Science and Engineering at Johns Hopkins University [31]. In addition to COVID-19 case and death aggregates, the dataset also includes variables from the University of Oxford COVID-19 Government Response Tracker (OxCGRT), which collects publicly available information on 20 indicators in different areas, such as containment policies, economic policies, and health policies in more than 150 countries since January 2020 [32]. Data are collected in real-time and from publicly available sources in each country. The OxCGRT dataset utilized in this paper was downloaded in March 2021.

**Table 1:** Open sources utilized in quantitative dataset.

VARIABLE	DESCRIPTION	SOURCE
iso_code	ISO 3166–1 alpha-3 –three-letter country codes	International Organization for Standardization
date	Date of observation	Our World in Data
total_cases	Total confirmed cases of COVID-19	COVID-19 Data Repository by the CSSE at Johns Hopkins University
new_cases	Total confirmed cases of COVID-19	COVID-19 Data Repository by the CSSE at Johns Hopkins University
total_deaths	Total deaths attributed to COVID-19	COVID-19 Data Repository by the CSSE at Johns Hopkins University
new_deaths	New deaths attributed to COVID-19	COVID-19 Data Repository by the CSSE at Johns Hopkins University
**Codebook for the Oxford Covid-19 Government Response Tracker NPIs**		
**COLUMN**	**DESCRIPTION**	**Coding**
stringency_index	All containment and closure policies (C1-C8) and one health system policy (H1)	Score of 0–100
government_response_index	All 20 policy indicators (C1-C8, and H1-H8), and economic relief (E1-E4) indicators	Score of 0–100
containment_health_index	All containment and closure (C1-C8) and health system (H1-H8) policies	Score of 0–100
c1_school_closing	Record closings of schools and universities	0—no measures 1—recommend closing or all schools open with alterations resulting in significant differences compared to non-Covid-19 operations 2—require closing (only some levels or categories, e.g., just high school, or just public schools) 3—require closing all levels Blank—no data
c1_flag	Binary flag for geographic scope	0—targeted 1- general Blank—no data
c2_workplace_closing	Record closings of workplaces	0—no measures 1—recommend closing (or recommend work from home) or all businesses open with alterations resulting in significant differences compared to non-Covid-19 operation 2—require closing (or work from home) for some sectors or categories of workers 3—require closing (or work from home) for all-but-essential workplaces (e.g., grocery stores, doctors) Blank—no data
c2_flag	Binary flag for geographic scope	0—targeted 1- general Blank—no data
c3_cancel_public_events	Record cancelling public events	0—no measures 1—recommend cancelling 2—require cancelling Blank—no data
c3_flag	Binary flag for geographic scope	0—targeted 1- general Blank—no data
c4_restrictions_on_gatherings	Record limits on gatherings	0—no restrictions 1—restrictions on very large gatherings (the limit is above 1000 people) 2—restrictions on gatherings between 101–1000 people 3—restrictions on gatherings between 11–100 people 4—restrictions on gatherings of 10 people or less Blank—no data
c4_flag	Binary flag for geographic scope	0—targeted 1- general Blank—no data
c5_close_public_transport	Record closing of public transport	0—no measures 1—recommend closing (or significantly reduce volume/route/means of transport available) 2—require closing (or prohibit most citizens from using it) Blank—no data
c5_flag	Binary flag for geographic scope	0—targeted 1- general Blank—no data
c6_stay_at_home_requirements	Record orders to "shelter-in-place" and otherwise confine to the home	0—no measures 1—recommend not leaving house 2—require not leaving house with exceptions for daily exercise, grocery shopping, and ’essential’ trips 3—require not leaving house with minimal exceptions (e.g., allowed to leave once a week, or only one person can leave at a time, etc.) Blank—no data
c6_flag	Binary flag for geographic scope	0—targeted 1- general Blank—no data
c7_movementrestrictions	Record restrictions on internal movement between cities/regions	0—no measures 1—recommend not to travel between regions/cities 2—internal movement restrictions in place Blank—no data
c7_flag	Binary flag for geographic scope	0—targeted 1- general Blank—no data
c8_internationaltravel	Record restrictions on international travel (this records policy for foreign travelers, not citizens)	0—no restrictions 1—screening arrivals 2—quarantine arrivals from some or all regions 3—ban arrivals from some regions 4—ban on all regions or total border closure Blank—no data
h1_public_information_campaigns	Record presence of public info campaigns	0—no Covid-19 public information campaign 1—public officials urging caution about Covid-19 2- coordinated public information campaign (e.g., across traditional and social media) Blank—no data
h1_flag	Binary flag for geographic scope	0—targeted 1- general Blank—no data
h2_testing_policy	Record government policy on who has access to testing Note: this records policies about testing for current infection (PCR tests) not testing for immunity (antibody test)	0—no testing policy 1—only those who both (a) have symptoms AND (b) meet specific criteria (e.g., key workers, admitted to hospital, came into contact with a known case, returned from overseas) 2—testing of anyone showing Covid-19 symptoms 3—open public testing (e.g., "drive through" testing available to asymptomatic people) Blank—no data
h3_contact_tracing	Record government policy on contact tracing after a positive diagnosis Note: we are looking for policies that would identify all people potentially exposed to Covid-19; voluntary Bluetooth apps are unlikely to achieve this	0—no contact tracing 1—limited contact tracing; not done for all cases 2—comprehensive contact tracing; done for all identified cases
h6_facial_coverings	Record policies on the use of facial coverings outside the home	0—No policy 1—Recommended 2—Required in some specified shared/public spaces outside the home with other people present, or some situations when social distancing not possible 3—Required in all shared/public spaces outside the home with other people present or all situations when social distancing not possible 4—Required outside the home at all times regardless of location or presence of other people
h6_flag	Binary flag for geographic scope	0—targeted 1- general Blank—no data
h8_protection_of_elderly_people	Record policies for protecting elderly people (as defined locally) in Long Term Care Facilities and/or the community and home setting	0—no measures 1—Recommended isolation, hygiene, and visitor restriction measures in LTCFs and/or elderly people to stay at home 2—Narrow restrictions for isolation, hygiene in LTCFs, some limitations on external visitors and/or restrictions protecting elderly people at home 3—Extensive restrictions for isolation and hygiene in LTCFs, all non-essential external visitors prohibited, and/or all elderly people required to stay at home and not leave the home with minimal exceptions, and receive no external visitors Blank—no data
h8_flag	Binary flag for geographic scope	0—targeted 1- general Blank—no data

This paper focuses on two policy indices calculated by the OxCGRT, the stringency index (SI) and the containment and health index (CHI). Table 2 shows which variables were included in each index. The SI records the strictness of closure and containment policies using 9 indicators, which include lockdown policies, primarily aimed at restricting certain behaviors, while the CHI includes all the variables from the stringency index and additional variables focused on mitigating the health consequences of COVID-19 (e.g., testing, use of facial coverings outside the home, and contact tracing) [32]. The methods and calculation of indices are described elsewhere by Hale et al [32]. Broadly, both indices aggregate the data of individual policy measures into a single number, between 0 to 100, that reflects the level of a government’s response along certain dimensions to measure the indicators upon which a government has acted, and to what degree.

**Table 2:** Non-pharmaceutical policy variables used in OxCGRT index calculation.

Variable	Description	SI	CHI
C1	Record closings of schools and universities	X	X
C2	Record closings of workplaces	X	X
C3	Record canceling public events	X	X
C4	Record limits on gatherings	X	X
C5	Record closing of public transport	X	X
C6	Record orders to "shelter-in-place" and otherwise confine to the home	X	X
C7	Record restrictions on internal movement between cities/regions	X	X
C8	Record restrictions on international travel (this records policy for foreign travelers, not citizens)	X	X
H1	Record presence of public info campaigns	X	X
H2	Record government policy on who has access to testing Note: this records policies about testing for current infection (PCR tests) not testing for immunity (antibody test)		X
H3	Record government policy on contact tracing after a positive diagnosis Note: Policies include only those that would identify all people potentially exposed to COVID-19		x
H6	Record policies on the use of facial coverings outside the home		x
H8	Record policies for protecting elderly people (as defined locally) in Long Term Care Facilities and/or the community and home setting		x

To examine the degree of implementation of NPIs relative to the four case windows, we used plots and descriptive statistics (means and standard deviations) stratified by the case windows. Indices were compared across case windows using analysis of variance (ANOVA) models with post-hoc comparisons (p-values adjusted using Tukey’s method). All analyses were performed using SAS statistical software version 9.4 (SAS Institute Inc., Cary, NC) [33].

### Ethical considerations

IRB exemption was granted on May 5, 2021, by the Boston University Medical Center Institutional Review Board (IRB number: H-41329). All key informant interviewees were informed that participation was voluntary. All participants provided verbal consent to being recorded before the start of the interview.

## Results

### Quantitative findings

It took Rwanda less time to surpass the first 100 cases (21 days) compared to Nigeria (30 days) and Zambia (43 days); however, it took Rwanda substantially longer to surpass 10,000 cases (379 days) than Nigeria and Zambia (152 and 232 days, respectively). All three countries experienced an exponential rise in cases after surpassing 1,000 cases of COVID-19.

An examination of SI scores from January 1^st^, 2021, until each country surpassed 10,000 cases, reveals several important differences across countries (Tables 3–5). In Rwanda, the average score of the SI from January 1^st^, 2020, until it surpassed 10,000 cases was 61.1 out of 100 (SD = 26.6, median = 73.2) indicating sustained moderate stringency on measures. In contrast, Nigeria and Zambia had average scores of 42.1 (SD = 37.1, median = 11.1) and 34.3 (SD = 22.3, median = 43.5), respectively. In Nigeria, a Tukey post hoc test shows the mean SI scores in case windows W_2_ (SI = 83.0) and W_3_ (SI = 84.6) were statistically significantly different (p<0.0001) when compared to W_0_ (SI = 6.2). However, there was no difference between the mean SI scores between W_2_ and W_3_ (p = 0.85), indicating stringency scores stayed relatively around the same level after the 100^th^ case. In contrast, in Zambia, the mean SI scores in case windows W_1_ (SI = 49.5), W_2_ (SI = 51.2), and W_3_ (SI = 47.4) were significantly different (p<0.0001) than W_0_ (SI = 5.6), indicating that Zambia’s stringency levels varied post-identification of the index case compared to pre-identification. However, there was no difference between the mean SI scores across case windows W_1_, W_2_, and W_3_, suggesting that the stringency levels in how the measures were implemented stayed relatively stable despite cases increasing. In Rwanda, mean SI scores in W_1_ (SI = 73.2) and W_3_ (SI = 70.3) were statistically significantly different (p<0.05) when compared to W_2_ (SI = 79.5). However, there was no difference between the mean SI scores between W_1_ and W_3_ (p = 0.53) suggesting that the stringency levels were similar in terms of which measures were implemented during W_1_ and W_3_.

**Table 3:** One-way analysis of variance of the stringency index score by case window, Nigeria.

				Tukey’s Post-hoc Comparisons			
Case Window	*n*	Mean	*SD*	W_0_	W_1_	W_2_	W_3_
**Pooled**	152	42.1	37.1	-	-	-	-
**W**_**0**_	58	6.2	5.1		<0.0001	<0.0001	<0.0001
**W**_**1**_	30	22.3	16.3	<0.0001		<0.0001	<0.0001
**W**_**2**_	26	83.0	0.6	<0.0001	<0.0001		0.85
**W**_**3**_	38	84.6	0.6	<0.0001	<0.0001	0.85	

Note: W_0_: 0 cases, W_1_: 1–100+ cases, W_2_: >101–1,000+ cases, W_3_: >1,001–10,000+ cases

*n*: number of days, *SD*: standard deviation

**Table 4:** One-way analysis of variance of the stringency index by case window, Rwanda.

				Tukey’s HSD Comparisons			
Case Window	*n*	Mean	*SD*	W_0_	W_1_	W_2_	W_3_
**Pooled**	379	61.1	26.6	-	-	-	-
**W**_**0**_	73	10.9	7.6		<0.0001	<0.0001	<0.0001
**W**_**1**_	21	73.2	25.6	<0.0001		0.04	0.54
**W**_**2**_	86	79.5	8.6	<0.0001	0.04		<0.0001
**W**_**3**_	199	70.3	7.5	<0.0001	0.54	<0.0001	

Note: W_0_: 0 cases, W_1_: 1–100+ cases, W_2_: >101–1,000+ cases, W_3_: >1,001–10,000+ cases

*n*: number of days, *SD*: standard deviation

**Table 5:** One-way analysis of variance of the stringency index by case window, Zambia.

				Tukey’s HSD Comparisons			
Case Window	*n*	Mean	*SD*	W_0_	W_1_	W_2_	W_3_
**Pooled**	232	34.3	22.3	-	-	-	-
**W**_**0**_	77	5.6	7.9		<0.0001	<0.0001	<0.0001
**W**_**1**_	43	49.5	14.7	<0.0001		0.87	0.63
**W**_**2**_	27	51.2	12.1	<0.0001	0.87		0.25
**W**_**3**_	85	47.4	5.1	<0.0001	0.63	0.25	

Note: W_0_: 0 cases, W_1_: 1–100+ cases, W_2_: >101–1,000+ cases, W_3_: >1,001–10,000+ cases

*n*: number of days, *SD*: standard deviation

There were also major differences between countries when examining the degree of implementation of the CHI which measured all eight containment and closure NPIs and eight health system NPIs (Tables 6–8). In Rwanda, the overall average score for the CHI was 58.5 (SD = 25.3, median = 71.4) (Table 6). In contrast, the average scores in Nigeria and Zambia were 38.3 (SD = 32.2, median = 16.7) and 31.1 (SD = 20.8, median = 40.5), indicating lower stringency and levels of implementation compared to Rwanda. When looking at differences in implementation between case windows, there were significant differences between the CHI scores across case windows in Nigeria indicating varying levels of implementation between the case windows. By comparison, in Rwanda, the mean containment and health index scores in case windows W_1_ (CHI = 65.3) and W_3_ (CHI = 68.7) were significantly different (p<0.001) compared to W_2_ (CHI = 74.7). However, there was no difference between the mean CHI scores in W_1_ and W_3_, indicating that the implementation of measures calculated in the CHI were similar during W_1_ and W_3_. Lastly, in Zambia, there was a statistically significant difference between the mean containment and health index scores in case windows W_1_ (CHI = 41.7), W_2_ (CHI = 47.8), and W_3_ (CHI = 45.3). However, there was no difference in the mean containment and health index scores between W_1_ and W_2_ compared to W_3_ (p>0.05), which again indicates similar implementation of the measures calculated in the containment and health index during W_1_, W_2_, and W_3_.

**Table 6:** One-way analysis of variance of the containment and health index by case window, Nigeria.

				Tukey’s Post-hoc Comparisons			
Case Window	*n*	Mean	*SD*	W_0_	W_1_	W_2_	W_3_
**Pooled**	152	38.3	32.2	-	-	-	-
**W**_**0**_	58	5.1	4.4		<0.0001	<0.0001	<0.0001
**W**_**1**_	30	26.5	13.8	<0.0001		<0.0001	<0.0001
**W**_**2**_	26	70.5	1.0	<0.0001	<0.0001		0.0065
**W**_**3**_	38	76.1	0.4	<0.0001	<0.0001	0.0065	

Note: W_0_: 0 cases, W_1_: 1–100+ cases, W_2_: >101–1,000+ cases, W_3_: >1,001–10,000+ cases

*n*: number of days, *SD*: standard deviation

**Table 7:** One-way analysis of variance of the containment and health index by case window, Rwanda.

				Tukey’s HSD Comparisons			
Case Window	*n*	Mean	*SD*	W_0_	W_1_	W_2_	W_3_
**Pooled**	379	58.5	25.3	-	-	-	-
**W**_**0**_	73	9.7	8.0		<0.0001	<0.0001	<0.0001
**W**_**1**_	21	65.3	21.0	<0.0001		<0.0001	0.24
**W**_**2**_	86	74.7	4.6	<0.0001	<0.0001		<0.0001
**W**_**3**_	199	68.7	6.2	<0.0001	0.24	<0.0001	

Note: W_0_: 0 cases, W_1_: 1–100+ cases, W_2_: >101–1,000+ cases, W_3_: >1,001–10,000+ cases

*n*: number of days, *SD*: standard deviation

**Table 8:** One-way analysis of variance of the containment and health index by case window, Zambia.

				Tukey’s HSD Comparisons			
Case Window	*n*	Mean	*SD*	W_0_	W_1_	W_2_	W_3_
**Pooled**	232	31.1	20.8	-	-	-	-
**W**_**0**_	77	3.6	5.1		<0.0001	<0.0001	<0.0001
**W**_**1**_	43	41.7	13.7	<0.0001		0.005	0.05
**W**_**2**_	27	47.8	7.8	<0.0001	0.005		0.43
**W**_**3**_	85	45.3	3.3	<0.0001	0.05	0.43	

Note: W_0_: 0 cases, W_1_: 1–100+ cases, W_2_: >101–1,000+ cases, W_3_: >1,001–10,000+ cases

*n*: number of days, *SD*: standard deviation

### Qualitative findings

A total of ten (n = 10) KIIs were conducted. Interviewees represented different agencies involved in the response including Ministries of Health, WHO AFRO, academic institutions, and non-governmental organizations. Several themes emerged regarding challenges in implementing NPIs from participants. The socioeconomic impact of the NPIs was a major theme. Other challenges included lack of adherence and compliance to measures and perceived severity of COVID-19 by the community. Several notable sub-themes were also identified. These are discussed below in more detail, along with illustrative statements.

Nearly all participants commented on the economic hardships that certain NPIs such as lockdowns and business closures have created.

*“… overall, the economy was affected, when businesses were closed, everywhere was closed, the economy was affected. Like we said earlier, there was also impact in accessing the services by the general population because of the movement restrictions. So, there are some of the things that were negatively impacted and has put the countries in a tight corner and made them start re-opening the economy and lifting some of those measures.”–Participant 1, WHO AFRO*

*“Businesses really suffered. All nightclubs are completely shut down now, they’re completely out of the picture. Many restaurants shut down. And right after the lockdown was lifted, restaurants became very expensive. They had to compensate for the last one full year. That has been a challenge.”–Participant 3, Rwanda*

Social determinants of health such as poverty, lack of access to water, overcrowding, and food insecurity were major sub-themes that emerged. Poverty was mentioned by nearly all participants as a major challenge in both implementation and adherence to NPIs. Without solutions to the underlying issues already facing citizens, implementing NPIs and expecting individuals to comply was difficult. The socioeconomic consequences of the public health and social measures that were put in place, especially containment and closure NPIs, cannot be adequately underscored. The protracted nature of the COVID-19 pandemic with no clear end in sight, even two years later, made it particularly difficult to continue to enact certain measures such as lockdowns and closure of businesses and schools. While NPIs play a great role in minimizing spread of disease, interviews with participants highlighted the complexities at play when trying to implement these measures.

*“…the challenges have been number one poverty [for groups of people]*. *There are several people in Africa, Rwanda included, that depend on day-to-day wages. Take the earnings of motorcycle drivers, taxi drivers, day-laborers, farmers. Quarantine for them has been a huge, huge blow, a huge blow. Vis-a-vi other African countries did not implement quarantine. I think it was only Uganda and Kenya that did so. So that has been a big challenge.”–Participant 3, Rwanda*

*“And then you also want to look at the fact that when you tell me wash your hands*, *and there’s no running water in your area, how are you going to wash your hands? Or when you say keep a safe distance and then you have a family of 6 living in a room, how are they going to keep a safe distance? And you have the whole block of single room apartments which is packed with so many people. Or in the marketplace, how do you keep distance in the market? So, some of the non-pharmaceutical interventions were not implementable because of the environment we are in.”–Participant 1, WHO AFRO*

An additional theme was perceived severity of disease. Several noted that media outlets, especially in the West, had emphasized that when COVID-19 reached Africa, it would create complete catastrophe. When this was not the case in the first and second wave, KII participants noted that citizens did not perceive COVID-19 to be a major threat and did not find the strict measures to be justified. KII participants mentioned that this view was further intensified by misinformation, and, in the case of Nigeria, major distrust in the government.

*“First of all, I’m sad to say that the major challenge was the people didn’t trust the government*. *Two, they didn’t believe there was COVID because people were not dying because the wrong impression was created that when COVID came to Africa it was going to kill everybody, and so when they didn’t see any dead bodies then they just assumed that there was no COVID. And there was a lot of fake information and things about COVID, which people took as truth.”–Participant 1, Nigeria*

*“…the number one challenge is the people’s perception of the disease itself because despite all the government did to make people understand how the disease is caught*, *you will hear people say it is a scam, that it is an attempt for government to just make money out of donors, that there’s no such disease as COVID… there was buy-in initially but because of these fear…the fear that the government cannot be trusted, because people think they had what they call “hidden agenda.”–Participant 2, Nigeria*

Lack of compliance and adherence to NPIs was also a major theme that emerged. In Zambia, the pandemic also coincided with a particularly tense election year, which brought its own challenges in terms of adherence to measures.

*“… it’s been an election year for Zambia*. *This is unique to us so we had political gatherings that I think needed time…they didn’t really adhere to us under our recommendations, or they said in principle they would accept that, and then just went ahead and did whatever they wanted to do. So, it was rather a challenging time. And we had large gatherings, political gatherings, that was a big challenge for us to manage adherence to COVID-19 regulations, as well as ensure that there is a democratic electoral process running side by side.”–Participant 1, Zambia*

Enforcement was a common theme among participants. In Rwanda, enforcement was mentioned as a success several times, and it was attributed to both the malleability of the Rwandese people as a population that respects its government and strict enforcement by the government.

*“Rwandans obey the rule of law. Obey their government*. *If there’s a policy, it’s respected, generally. Even without an enforcer, without the involvement of the police, of law enforcement, it is generally accepted. That’s the main thing. The second thing is they’re very strict. Walking without a mask is I think $30. I’ve seen several people getting penalized right in front of my eyes in Kigali. So, they don’t joke around. If you are caught past the curfew, you will be taken to the national stadium, you’ll be kept there throughout the night—that’s punishment in addition to $100 payment.”–Participant 3, Rwanda*

*“Like everyone had to be home at 7pm*. *Right now, the time is 7:45pm and if you go on the road, you won’t find anybody. Just that move of respecting the guidelines and the level of cooperation is why I think we’re successful.”–Participant 1, Rwanda*

*“…the enforcement piece I could say that there were radio talks about any of the measures that were put in place by the government and local entities were in charge of making sure these measures are respected by the general population*.*”–Participant 2, Rwanda*

Whereas enforcement was a success in Rwanda, enforcement was a major challenge in Nigeria and Zambia. KII participants noted that the length and protracted nature of the response have had major consequences in terms of compliance and adherence to several public health and social measures that were being implemented at various points throughout the pandemic, not only by the community but some KIIs mentioned government figures themselves were not adhering to precisely the measures they were responsible for enforcing.

*“…there were not really [consequences for lack of adherence]* …well they put it there…but complying with the law and enforcing is one thing…on a few cases here and there they did. But it didn’t last. In about a week or two everybody had forgot, and they went back…even the guy who is supposed to be enforcing it is not wearing a mask *laughs*”–Participant 1, Nigeria

*“For places of worship, they are now back to their old*, *crowded form despite the regulations. Because the latest law was no gathering of 50 or more people, but I was in church on Sunday, and I think we were more than 2,000 in that gathering so that’s how it is. So, there is no consistency or uniformity in the enforcement. Like every state or local government can do whatever they felt like implementing.”–Participant 2, Nigeria*

*“…in some of the large density populations in the peri urban areas, if you went there*, *they would just see blatant disregard of the regulations despite posters being around or motor vehicles with the speakerphone going around announcing what to do. You would essentially see nobody wearing for instance a mask, and that just shows you the defiance levels people had, and because we didn’t really have people going around arresting you for not putting on a mask for instance, so that was some of the challenges we saw.”–Participant 1, Zambia*

In the opinion of KII participants, political structures and dynamics also played a factor as a barrier in the implementation of NPIs. In countries like Nigeria and Zambia, where there is a decentralized government, enforcement was much more difficult than in a country like Rwanda, which has a very centralized government.

*“Over time, there was no consistency in the enforcement and no uniformity between States*. *Some States tended to take it more seriously than other States. But on the part of the Federal Government of Nigeria, the government had been the one really wielding the big stick. The States were left to do what they felt like doing because at first when the [federal] government tried to send down regulations right from the country’s capital, some State governments saw it as [an] affront to their own rights because the body governance and the president were elected so they didn’t like the idea of a [federal] government official trying to decide what happens at the State level. So, the States were left to do what they wanted to do.”–Participant 2, Nigeria*

*“It also depends on the political structure of the country*. *Nigeria is a very complex country where it has federal [territories] and states. The states have very much power in terms of dealing with the local issues, so the application and implementation of these measures at the local level was a little bit not much uniform in the country. Whereby in countries like Rwanda, it’s most centralized, of course with good leadership, the implementation of these measures was very much uniform across the country.”–Participant 2, WHO AFRO*

## Discussion

Utilizing two indices of the OxCGRT database, we were able to examine the degree in which NPIs were implemented. While the quantitative data show the differences in the indices, the qualitative data allow for a deeper understanding of the nuances of public health and social measures in the three countries. In addition, the qualitative data provide rich information on the barriers and facilitators of implementing NPIs during the pandemic. As key informants noted, enforcement of NPIs was met with resistance and noncompliance in countries where governmental authority was weak or contested, or misinformation was high [34–37]. Similarly, physical, or social distancing measures were also difficult to enforce and implement. Aside from the high population density in many communities in Africa, social interaction is a key aspect of life. In urban areas in Africa, public transportation systems are often overcrowded, dense shanty towns and informal settlements are part of the physical infrastructure, and many people do not have the luxury to self-isolate even if they are positive, as many homes face overcrowding. For example, Makoko in Lagos, Nigeria has 300,000 people whose homes are built on stilts in a lagoon [35]. In rural areas, many households share sanitation facilities and have only access to water from a communal tap. Ekumah et al. (2020) used demographic and health survey data from 25 countries in sub-Saharan Africa to explore how vulnerability to COVID-19 was affected by access to basic necessities (sanitation, water, and food) within a household [38]. They found that 46% of sampled households (except South Africa) lacked access to any of these three basic necessities, and only 8% had access to all three [38]. Thus, physical distancing measures, including shelter-in-place measures, were unrealistic in overcrowded areas with inadequate sanitation as pointed out by KIIs.

In addition to several long-term effects of implementing NPIs, timing plays a crucial role in the implementation of NPIs. Several KIIs including those at WHO AFRO pointed out early measures undertaken by countries. However, experts may find it challenging to determine the optimal time to implement different interventions. If governments wait too long, this may lead to the proliferation of disease at a rapid rate and overwhelm health systems quickly. Consequently, the roll-out of NPIs that are too premature or uniform across an entire country may also increase the risk of a “second wave” of infections once the initial measures are halted or pandemic fatigue sets in [39, 40]. The implementation of NPIs, especially over a prolonged time period, can have significant detrimental consequences in terms of social and economic costs as was the case in Nigeria, Rwanda, and Zambia. While NPIs are generally effective in reducing the burden of disease and alleviating pressure on health infrastructures, studies have found that a longer duration of NPI implementation may have consequences such as increased unemployment, economic hardship, and social disruption [11]. Resource-poor settings are at an especially increased risk, with some data showing income reduction as great as 70% and reduction in consumption expenditure by 30% [11, 41, 42]. The Africa Research, Implementation Science, and Education (ARISE) Network conducted a telephone survey in Burkina Faso, Ethiopia, and Nigeria between July and November 2020 to understand COVID-19 knowledge, attitudes, practices, and their impacts on health, nutrition, and education [43, 44]. The education sector was profoundly affected by school closures. Food shortages and insecurity were rampant across the three sites in the study. Consumption of a range of staple foods in all three countries declined, while the prices of staples, legumes, vegetables, fruit, and animal-source foods rose [43, 45]. Additionally, with increased unemployment and decreased crop production, respondents described reductions in general food intake and dietary diversity [43, 45].

Compliance is also a major factor in NPI implementation. NPIs are dependent on enforcement and citizens’ willingness to comply with the measure. Compliance generally wanes the longer measures are in effect [10]. Poverty and economic dislocation also reduce compliance especially with NPIs focused on containment (i.e., shelter-in-place) which again was supported by KIIs in our study [46]. In Rwanda, compliance with public health measures, including mask-wearing, was governed strictly by police and an anti-corona task force. Anecdotal reports detailed police pulling over cars with unmasked drivers and/or passengers, hand-washing stations and sanitizer dispensers were monitored for use before entering businesses, and arrests were made of those violating the country-wide curfew [47]. The strictness with which measures were implemented in Rwanda was supported by claims made by KIIs. This was also supported by the quantitative data where the pooled average SI and CHI scores for Rwanda were much higher than that of Nigeria and Zambia. Additionally, despite a robust public information campaign to dispel misinformation, many residents in Nigeria did not initially adhere to the recommendations aimed at reducing the spread of COVID-19. This caused high tension between the civilians and armed forces who became violent when trying to enforce certain protocols [48, 49]. To enforce lockdown and curfew measures, policy, paramilitary, and military personnel were deployed to various areas in the country. Among the challenges in this implementation were persistent corruption and political distrust [48, 49]. There were also multiple reports of unlawful use of force and misconduct of the Nigerian police while enforcing COVID-19 measures [50, 51]. Implementation and enforcement were marred by a lack of compliance from the public, which limited the outcomes of the government response to COVID-19 by weakening the impact of travel restrictions and lockdown measures on the slowing down of COVID-19 spread in the country which was also supported by KIIs.

The study has several limitations. First, given the fluidity of the COVID-19 pandemic, external factors such as new SARS-CoV-2 variants of concern, holiday season, etc., could have affected the degree of implementation. For example, Nigeria reached 10,000 cases in May 2020 while Rwanda reached that point in January 2021. We also acknowledge that case counts in virtually every country were severe underestimations. At the beginning of the pandemic, case counts were affected due to lack of access to accurate diagnostics, weak surveillance systems, and case definitions. Thus, case counts in this paper are likely to be underestimates of the true burden of disease in all three countries. The state of the pandemic and global guidance had changed significantly in between that time. There are also specific limitations in the OxCGRT dataset itself. The dataset does not measure implementation or compliance, nor does it provide subnational measures for almost all countries apart from adding a flag denoting whether the restriction was national or subnational [32]. Thus, our nation-focused analysis may miss some variation of policies implemented at the sub-national level. Additionally, our sample size of KIIs is relatively small, therefore there may be other diverse opinions about what worked and what did not during NPI implementation, that were not captured here.

To the best of our knowledge, this study is the first to focus on understanding the degree of implementation and facilitators and barriers to enforcement of NPIs in sub-Saharan Africa. There continues to be limited evidence about the impact of NPIs on the progression of the pandemic in Africa. Existing research on the impact of NPIs on COVID-19 focuses on high-income countries in Europe and Asia, who were heavily affected at the beginning of the global pandemic, not on African countries. This paper aims to add to the body of knowledge on infectious disease mitigation efforts while informing public health decision-making and policies and programs at the regional and country level in Africa.

## Conclusion

One of the most important lessons learned, and a key recommendation for continued and/or future implementation of NPIs, is the early engagement of communities in a pandemic or outbreak response. The success of NPIs is largely dependent upon the willingness and compliance of citizens to adopt control measures. Ideally, community engagement should begin during the preparedness stage. Listening to communities, understanding their concerns, and providing them with the right information will all be critical in ensuring high compliance and building trust. The latter is especially important as we found through key informants that government distrust and misinformation served as a barrier to implementation. Secondly, a risk-based approach should be used to implement containment and closure measures especially those that restrict people’s behaviors. A risk-based approach to implementation was strongly advocated by WHO AFRO personnel consulted during this analysis. A risk-based approach utilizes surveillance and epidemiological data to inform experts where measures should be implemented, rather than the implementation of blanket measures (e.g., national lockdown). For example, containment and closure measures can be targeted at communities with high rates of transmission rather than at the national level. Lastly, providing economic and social support to communities is crucial, especially during the implementation of measures that include the closure of schools and businesses and movement restrictions that limit the ability of individuals to access education or to leave their homes to earn a living. For example, innovative financial strategies in Rwanda included supporting workers and vulnerable persons through food relief efforts to the hardest-hit households living in Kigali and other urban centers, zero charges on mobile money transfers, and removing the maximum transaction limit on mobile payments. These interventions were able to alleviate some of the economic consequences of lockdowns and closure of businesses for the Rwandese people. Given the success of engaging communities during early stages of the response, implementing measures using a risk-based approach, and providing social and economic support to citizens, entities like Africa CDC and WHO should consider incorporating these strategies in guidance offered to all Member State.
